# gga-mir-133a-3p Regulates Myoblasts Proliferation and Differentiation by Targeting *PRRX1*

**DOI:** 10.3389/fgene.2018.00577

**Published:** 2018-12-04

**Authors:** Lijin Guo, Weiling Huang, Biao Chen, Endashaw Jebessa Bekele, Xiaolan Chen, Bolin Cai, Qinghua Nie

**Affiliations:** ^1^Department of Animal Genetics, Breeding and Reproduction, College of Animal Science, South China Agricultural University, Guangzhou, China; ^2^Guangdong Provincial Key Lab of Agro-Animal Genomics and Molecular Breeding and Key Lab of Chicken Genetics, Breeding and Reproduction, Ministry of Agriculture, Guangzhou, China

**Keywords:** gga-mir-133a-3p, *PRRX1*, myoblast, proliferation, differentiation

## Abstract

Non-coding RNAs play a regulatory role in the growth and development of skeletal muscle. Our previous study suggested that gga-mir-133a-3p was a potential candidate for regulating myoblast proliferation and differentiation in skeletal muscle. The purpose of our study was to reveal the regulatory mechanism of gga-mir-133a-3p in the proliferation and differentiation of chicken myoblasts. Through the detection of cell proliferation activity, cell cycle progression and EdU, we found that gga-mir-133a-3p can significantly inhibit the proliferation of myoblasts. In the process of myogenic differentiation, gga-mir-133a-3p is up-regulated, while gga-mir-133a-3p can significantly promote the up-regulation of differentiation-related muscle-derived factors, indicating that gga-mir-133a-3p can promote the differentiation of myoblasts. Validation at the transcriptional level and protein level proved that gga-mir-133a-3p can inhibit the expression of *PRRX1*, and the dual-luciferase assay also showed their direct targeting relationship. Correspondingly, *PRRX1* can significantly promote myoblast proliferation and inhibit myoblast differentiation. In our study, we confirmed that gga-mir-133a-3p participates in the regulation of proliferation and differentiation of myoblasts by targeting *PRRX1*.

## Introduction

Skeletal muscle is the most important part of the body of the meat-producing animals, and its growth and development are closely related to the meat production. Skeletal muscle growth and development is a very complex biological process, subject to many signal pathways and factors of regulation (Bassel-Duby and Olson, 2006; Schiaffino et al., 2007). Myogenesis is a process in which somatic cells undergo a series of processes of proliferation, migration, differentiation, and ultimately formation of muscle tissue during embryonic development (Perry and Rudnick, 2000; Rehfeldt et al., 2000). The coordinated regulation of myogenic renewal is a major part of skeletal muscle growth and development, including myoblast proliferation, differentiation, apoptosis, and fusion (Sartorelli and Fulco, 2004). The process of myoblast proliferation and differentiation is also regulated by many signaling pathways and regulatory factors, including DNA, non-coding RNAs (ncRNAs), and peptides.

Non-coding RNAs are mainly composed of microRNAs (miRNAs), long-chain non-coding RNAs (lncRNAs), circular RNAs (circRNAs). The function and role of non-coding RNAs in the myogenic proliferation and differentiation had been characterized (Wei et al., 2013; Jia et al., 2017; Liang et al., 2018). The presence of circRNAs and lncRNAs have been confirmed in chickens, and some studies showed that circRNAs and lncRNAs can regulate the proliferation and differentiation of myoblast (Kallen et al., 2013; Ballarino et al., 2015; Wang et al., 2015; Zhuang et al., 2015; Ouyang et al., 2017; Panda et al., 2017; Wei et al., 2017). MicroRNAs are endogenous non-coding RNA molecules with a sequence of 20∼22 nt, which were identified to be the most important non-coding RNAs (Lin et al., 2012). MicroRNAs can regulate expression of target protein-coding genes at the transcription level by binding the 3′ untranslated region (3′UTR) of the target mRNAs (Lin et al., 2009). In addition, miRNAs can also inhibit translation and regulate the splicing process by binding to the 5′untranslated region (5′UTR) and the coding region of the target gene (Grey et al., 2010; Hausser et al., 2013; Chung et al., 2017; Agarwal et al., 2018; Zhang et al., 2018). The role of miRNAs in the proliferation and differentiation of myoblasts has also been characterized (Bartel, 2004; Luo et al., 2014; Shenoy and Blelloch, 2014). The study has shown that non-coding RNAs are critical for controlling myogenesis, including myoblast proliferation, differentiation, through the interaction of myogenic-related genes (Lewis et al., 2005). A recent study indicated that mir-200a can inhibit the proliferation and differentiation of myoblasts in the skeletal muscle of chicken (Jiang et al., 2017). In a similar vein, another study showed that mir-16-5p regulates p53 signaling pathways to affect myoblast proliferation apoptosis and differentiation by target to *SESN1* (Cai et al., 2018). These results further identify the importance of microRNAs in the growth and development of chicken skeletal muscles.

Gga-mir-133a expressed differently in breast muscle of fast-growing and slow-growing broilers, and it was the most abundant miRNA in the breast muscle sequencing libraries (Ouyang et al., 2015). Similarly, based on our previous study (accession number GSE100321) (Jebessa et al., 2018), gga-mir-133a-3p was differentially expressed in the leg muscles between the E11 and E16 XingHua chickens. The mir-133 family includes two members, mir-133a and mir-133b, as encoded by four loci in chick. Gga-mir-133a-3p is the mature miRNA which derived from two precursor miRNAs (gga-mir-133a-1 and gga-mir-133a-2), with a mature sequence of 22nt. In the previous studies, mir-133 had the characteristic of muscle-specific expression, it regulated the process of proliferation and differentiation of skeletal muscle cells through coordinated transcriptional regulation of muscle growth, regulation of alternative splicing regulators, and regulation of growth hormone pathway regulatory factors (Chen et al., 2006; Rao et al., 2006). It has been shown that mir-133 can enhance the proliferation of mouse myoblasts by targeting *SRF* (Chen et al., 2006). Another study has shown that mir-133 is upregulated during myoblast differentiation (Huang et al., 2011), which suggested that gga-mir-133a-3p may also be involved in myogenic differentiation of skeletal muscle as a member of mir-133 family. Gga-mir-133 can stabilize the myogenic differentiation program in the chick embryo (Goljanek-Whysall et al., 2014). Myoblasts first proliferate and differentiate into myocytes, and then myocytes fusion into myotubes and eventually mature into muscle fibers, which is the most important process of skeletal muscle growth. In mammals or poultry, the numbers of muscle fibers have been fixed before birth or within 1 week of birth (Smith, 1963; Li et al., 2015), therefore, the growth of skeletal muscle before birth mainly depends on the proliferation and differentiation of myoblasts. In our previous study (accession number GSE100321), gga-mir-133a-3p was differentially expressed in the leg muscles between the E11 and E16 XingHua chickens, which indicated that it may participate in the proliferation and differentiation of myoblasts. However, the study of gga-mir-133a-3p in poultry is rare, especially concerning of skeletal muscle growth and development, it is still very unclear.

*PRRX1* (paired related homeobox 1), a member of the Homeobox (*HoX*) genes, is an important gene with a higher genetic level and involves in formation of cartilage and bone, cell differentiation and morphological development (Martin et al., 1995; Ten et al., 1998; Higuchi et al., 2014). The *PRRX1* gene was highly expressed during the embryonic stage and had an important influence on the development and differentiation of embryos and the development of limb structures and organs in mice (Higuchi et al., 2013). The *PRRX1* gene, was mainly involved in the development of bone and cardiovascular system in the embryo, such as limbs, craniofacial, cartilage and arterial tubes (White et al., 2003; Yoshida et al., 2004). In addition, the *PRRX1* gene was also differentially expressed in the leg muscles between the E11 and E16 XingHua chickens in our previous study (accession number: GSE91060) (Jebessa et al., 2018). However, the *PRRX1* gene is rarely studied in the myogenesis of chicken skeletal muscle, and whether its role in the growth and development of skeletal muscle is regulated by microRNAs, it is still unknown.

In this study, we analyzed the expression pattern of gga-mir-133a-3p in skeletal muscle differentiation of fetal stage chickens and characterized its regulation on the growth and development of chicken skeletal muscles. In order to investigate the regulatory mechanism of gga-mir-133a-3p on the proliferation and differentiation of myoblasts, we conducted a targeted study of the *PRRX1* gene and demonstrated their direct target relationship. We also explored the effect of the *PRRX1* gene in the proliferation and differentiation of myoblasts. Our result demonstrated that gga-mir-133a-3p inhibits myoblast proliferation and promotes myoblast differentiation by targeting *PRRX1*.

## Materials and Methods

### Ethics Statement

The animal experiments in this study were approved by the Animal Care Committee of South China Agricultural University (Approval number: SCAU#0014). The experiments were conducted according to the rules and policies formulated by the committee and in accordance with the Animal Protection Law of the People’s Republic of China.

### Cell Culture

Chicken primary myoblasts (CPMs) were isolated from the leg muscles of 11- embryo-age chicken. The leg muscle was separated from the bone and crushed into pieces smaller than 1 mm^3^, and shaken in a shaker with 0.25% trypsin (Invitrogen, United States) at 37°C for 20 min. An equal amount of the RPMI 1640 Medium (Gibco, United States) with 20% fetal bovine serum (Gibco, United States) and 0.2% penicillin/streptomycin (Invitrogen, United States) was used to stop digestion. After centrifugation at 2,000 × *g* at room temperature, the supernatant was discarded and the cells were resuspended in the RPMI 1640 Medium with 20% fetal bovine serum and 0.2% penicillin/streptomycin and inoculated into a cell culture flask. Incubate in the incubator for 40 min and transfer the supernatant to a new flask. This step is repeated once. The culture of CPMs was performed using the RPMI 1640 Medium with 20% fetal bovine serum and 0.2% penicillin/streptomycin in a 37°C, 5% of CO_2_ incubator. Different media were used to induce differentiation of myoblasts, consisting of 2% horse serum (Hyclone, United States) and 0.2% penicillin/streptomycin (Invitrogen, United States) in 1640 medium. Quail muscle clone 7 (QM-7) cells were cultured in Medium 199 basic (Gibco, United States) with 10% fetal bovine serum (Gibco, United States), 10% tryptose phosphate broth solution (Sigma, United States) and 0.2% penicillin/streptomycin (Invitrogen) in a 37°C, 5% CO_2_ incubator. Dermal fibroblast cells (DF-1) were cultured with DMEM (Gibco, United States) with 10% fetal bovine serum (Gibco, United States) and 0.2% penicillin/streptomycin (Invitrogen, United States).

### Reverse Transcription and RT-PCR Analysis

Total RNA from tissues or cells was extracted following to the standard protocol of chloroform method. The tissue was ground and 1 mL of TRizol cell lysate (Invitrogen, Carlsbad, CA, United States) was added and incubated for 5 min at room temperature. Then add 0.2 mL of chloroform (Damao, Tianjin, China), shake for 15 s, incubate for 5 min at room temperature, centrifuge at 12,000 × *g* for 15 min at 4°C. After centrifugation, the supernatant was transferred to a new tube, and 0.5 mL of isopropanol (Damao, Tianjin, China) was added and shaken for 15 s. After incubation for 30 min at -80°C, centrifugation was performed at 4°C for 12,000 × *g* for 10 min. The supernatant was discarded and washed with 75% anhydrous ethanol (Damao, Tianjin, China). After centrifugation at 7,500 × *g* at 4°C for 5 min, the supernatant was discarded and dried for 4 min and diluted with 25 μL of RNAfree water (SangonBio, Shanghai, China). The reverse transcription kit (Toyobo, Japan) was used to perform RNA reverse transcription and synthesize double-stranded cDNA. The expression of mRNA and miRNA were detected by RT-PCR with Bio-rad CFX96 instrument (Bio-Rad, United States) using iTAPTM universal SYBR GREEN superMIX (Bio-Rad, United States). The internal controls used in the experiments for quantifying microRNA and mRNA were U6 and GAPDH, respectively. The primers used during the study are listed in Table 1. Relative expression of miRNA and mRNAs were calculated using the 2^-ΔΔCT^ method (ΔCT = CT_target gene_-CT_reference gene_, ΔΔCT = ΔCT_target gene_-ΔCT_control gene_). Three replicates were performed for RT-PCR analysis.

**Table 1:** Primers used for RT-PCR.

Gene	Primer sequences (5′–3 ′)	Size	Annealing	Accession number	Genomic coordinates
name		(bp)	temperature (° C)		
*PRRX1*	F: ACTAGAGAGGGTCTTTGAGAG	183	58.1	NM_001277724.1	8: 5223330–5223350
	R: GAGTAGGATTTGAGCAGAGAG				8: 5222595–5222615
*MYOD*	F: GCTACTACACGGAATCACCAAAT	200	58	NM_204214.2	5: 12397529–12397551
	R: CTGGGCTCCACTGTCACTCA				5: 12398326–12398345
*MYOG*	F: CGGAGGCTGAAGAAGGTGAA	320	58	NM_204184.1	26: 981493–981512
	R: CGGTCCTCTGCCTGGTCAT				26: 978865–978883
*MYHC*	F: CTCCTCACGCTTTGGTAA	213	58	NM_001319304.1	18: 394934–394938, 395058–395070
	R: TGATAGTCGTATGGGTTGGT				18: 394555–394574
*GAPDH*	F: TCCTCCACCTTTGATGCG	146	50-65	NM_204305.1	1: 77622636–77622653
	R: GTGCCTGGCTCACTCCTT				1: 77623118–77623125

### RNA Oligonucleotides and Plasmids Construction

Gga-mir-133a-3p mimic, gga-mir-133a-3p inhibitor, mimic NC, inhibitor NC, small interfering RNA (siRNA) and siRNA negative control used in this study were designed and synthesized (RiboBio, Guangzhou, China). The information of oligonucleotides sequences in the study is listed in Table 2.

**Table 2:** Oligonucleotide sequences in this study.

Sequence name	Sequences (5′–3′)	Genomic coordinates
gga-mir-133a-3p mimic	UUGGUCCCCUUCAACCAGCUGU	20: 8135589–8135600
Mimic NC	UUUGUACUACACAAAAGUACUG	X: 11791317–11791338 (Caenorhabditis elegans)
gga-mir-133a-3p inhibitor	ACAGCUGGUUGAAGGGGACCAA	Reverse complementary sequence
Inhibitor NC	UUUGUACUACACAAAAGUACUG	Random sequence
siRNA	CTGAACCGCTCGAGTGACA	8: 5217229–5217247

The complete CDS sequence of *PRRX1* was amplificated by PCR and was cloned into expression vector, pcDNA3.1 (Promega, United States). The restriction sites we chose were HindIII and XhoI. The segment sequence of the *PRRX1* 3′UTR that contained the putative gga-mir-133a-3p binding sequence was amplified and was subcloned into HindIII and XhoI sites in the pmirGLO dual-luciferase reporter vector (Promega, United States). The *PRRX1*-3′UTR mutant plasmid was obtained by converting the binding site of gga-mir-133a-3p from GGGACCA to CCCTAAT. PCR amplification of the mutants and DPNI digestion removed the parental DNA. The primers used to construct the plasmids were listed in Table 3.

**Table 3:** Primers used for plasmids construction.

Primer name	Primer sequences (5′–3′)	Size	Annealing	Genomic	coordinates
		(bp)	temperature (°C)	
pcDNA3.1+*PRRX1*	F: CCC**AAGCTT**ATGGCGTCCAGCTATGCCCA	738	63	8: 5248604–5248623
	R: CCG**CTCGAG**TTAATTGACTGTGGGCACTTG			8: 5217429–5217449
pmirGLO-*PRRX1*-3′UTR-WT	F: CCC**AAGCTT**AAAAAAATTAAAAAAGCC	100	50	8: 5217292–5217309
	R: CCG**CTCGAG**CAAAAGGAAGGAGGAACC			8: 5217210–5217227
pmirGLO-*PRRX1*-3′UTR-MT	F: AGAACTGAACATTCCCTAATAAGCGGGAGCAAA	2,771	65	8: 5217248–5217280
	R: TCTTGACTTGTAAGGGATTATTCGCCCTCGTTT			8: 5217248–5217280

*Bold sequence represents restriction enzyme recognition sequence*.

### MiRNA Targets Prediction and RNAhybrid Detection

MiRDB was used to predicted target genes of gga-mir-133a-3p (miRDB^1^). RNAhybrid detection was used to calculate the combined minimum free energy (MFE) of gga-mir-133a-3p and the 3′UTR of *PRRX1* to determine the binding stability of the duplex (RNAhybrid^2^).

### Cell Transfection

When the cells grow to 70% confluence, Lipofectamine 3000 Reagent (Invitrogen, United States) was used to perform transient transfections following its protocol. The concentrations used for plasmid transfections were as follow: 2.5 μg/well for 6-well plate; 1 μg/well for 12-well plate; 0.5 μg/well for 24-well plate; 0.25 μg/well for 48-well plate; 0.1 μg/well for 96-well plate. For mimics, inhibitors and siRNA, a concentration of 20, 100, and 100 nM were used, respectively.

### Dual-Luciferase Reporter Assay

After co-transfection of pmirGLO-*PRRX1*-3′UTR-WT or pmirGLO-*PRRX1*-3′UTR-MT with gga-mir-133a-3p mimic or mimic NC for 48 h, respectively, firefly and Renilla luciferase activities were detected using a Dual-GLO Luciferase Assay System Kit (Promega, United States) according its instruction. Multi-function microplate reader (Biotek, United States) was used to detect the firefly luciferase and Renilla luminescence activities.

### CCK-8 (Cell Counting Kit 8) Assay

Cell Counting Kit 8 assay was used to detect cell proliferation. CPMs or QM-7 cells were seeded in a 96-well plate and cultured in mediums. After transfection, cell proliferation was monitored at 12, 24, 36, and 48 h using a TransDetect CCK Kit (TransGen Biotech, Beijing, China), following the manufacturer’s protocol. Add 10 uL CCK solutions to each well and incubate for 1 h at 37°C in a 5% CO_2_ cell incubator. Then absorbance was measured using a Model 680 Microplate Reader (Bio-Rad, United States) by optical density at a wavelength of 450 nm.

### EdU (5-Ethynyl-2′-Deoxyuridine) Cell Proliferation Assay

For EdU cell proliferation assay, cells were seeded in 96-well plates. After transfection for 48 h, the cells were incubated at 37°C for 2 h in the presence of 50 μM EdU (RiboBio, Guangzhou, China). The cells were then fixed 4% paraformaldehyde for 30 min and neutralized using 2 mg/mL glycine solution for 5 min. Then the cells were permeabilized by 0.5% Triton X-100 for 10 min. The solution with EdU (RiboBio, Guangzhou, China) was added and the cells were incubated at room temperature for 30 min. Subsequently, the cells were permeabilized 3 times with 0.5% Triton X-100 for 10 min each time and then added with methanol for 5 min to reduce the dye background. The nuclear stain Hoechest 33342 was then added and incubation for another 30 min. Three randomly selected interfaces were captured using a fluorescence microscope (DMi8; Leica, Germany) to visualize the number of EdU-stained cells.

### Flow Cytometric Analysis

CPMs and QM-7 cells were seeded in 12-well plates. Transfection was performed when the cell density reached 50%. After 48 h of culture, cells were digested by trypsin, and then ceased the digestion using DMEM with 10% FBS, transfer the lysate to 1.5 mL micro-centrifuge tubes and centrifuge at 2,000 × *g* for 5 min at 4°C. After the centrifugation, discarded the supernatant and then washed them with 1 mL PBS and centrifuged at 2,000 × *g* for 5 min at 4°C, the step was repeated twice, and then fixed the cells in 70% ethanol overnight at -20°C. The fixed cells were stained with propidium iodide (Beyotime, Shanghai, China). And then the cells were incubated at 37°C for 30 min in the dark. CytoFLEX FCM (Beckman, United States) was used to flow cytometric analysis and the data was analyzed using FlowJo7.6 software.

### Western Blot

Ice-cold radio immunoprecipitation (RIPA) lysis buffer (Beyotime, Shanghai, China) with 1 mM phenylmethyl sulfonyl fluoride (Beyotime, Shanghai, China) was used in cell protein extraction. Proteins were separated by 10% SDS-PAGE, transferred to a nitrocellulose membrane (Maidstone, United Kingdom, Whatman), and then detected using antibodies according to standard procedures. The antibodies used for Western blots and their dilutions were as follows: Anti-*PRRX1* antibody (Abcam, United Kingdom) 1: 1,000 and GAPDH (Boster, Wuhan, China) 1: 2,000. Finally, a secondary antibody (Boster, Wuhan, China) containing horseradish peroxidase chain reaction (HRP) anti-rabbit IgG antibody (Boster, Wuhan, China) was incubated in a 1: 5,000 dilution.

### Immunofluorescence

For immunofluorescence, cells were seeded in 24-well plates. After transfection for 48 h, the cells were washed twice with PBS for 5 min and then fixed with 4% paraformaldehyde for 30 min. The cells were permeabilized by the addition of 0.1% TritonX-100 for 15 min and blocked with goat serum for 30 min. The cells were spiked with *MYHC* (B103; DHSB, United States; 0.5 μg/mL) and incubated overnight at 4°C. Subsequently, Goat Anti-Mouse IgG/FITC antibody (BS0296G; Bioss, China) was added and incubated for 1 h at room temperature. The DAPI was used for staining of the nucleus for 5 min. Fluorescence microscopy (DMi8; Leica, Germany) was used to capture images. The ImageJ software (National Institutes of Health) was used to measure the area of cells that were resistant to *MYHC*, and the calculated value is the percentage of the total area of the myotubes that occupy the image area.

### Statistical Analysis

All data were mean ± standard deviation (S.E.M.), and there were at least three independent experiments for each treatment. The *p*-values were calculated using an unpaired two-sample *t*-tests (Student’s *t*-tests), with a significant difference (*p* < 0.05).

## Results

### gga-mir-133a-3p Inhibits Myoblast Proliferation

**FIGURE 1:**
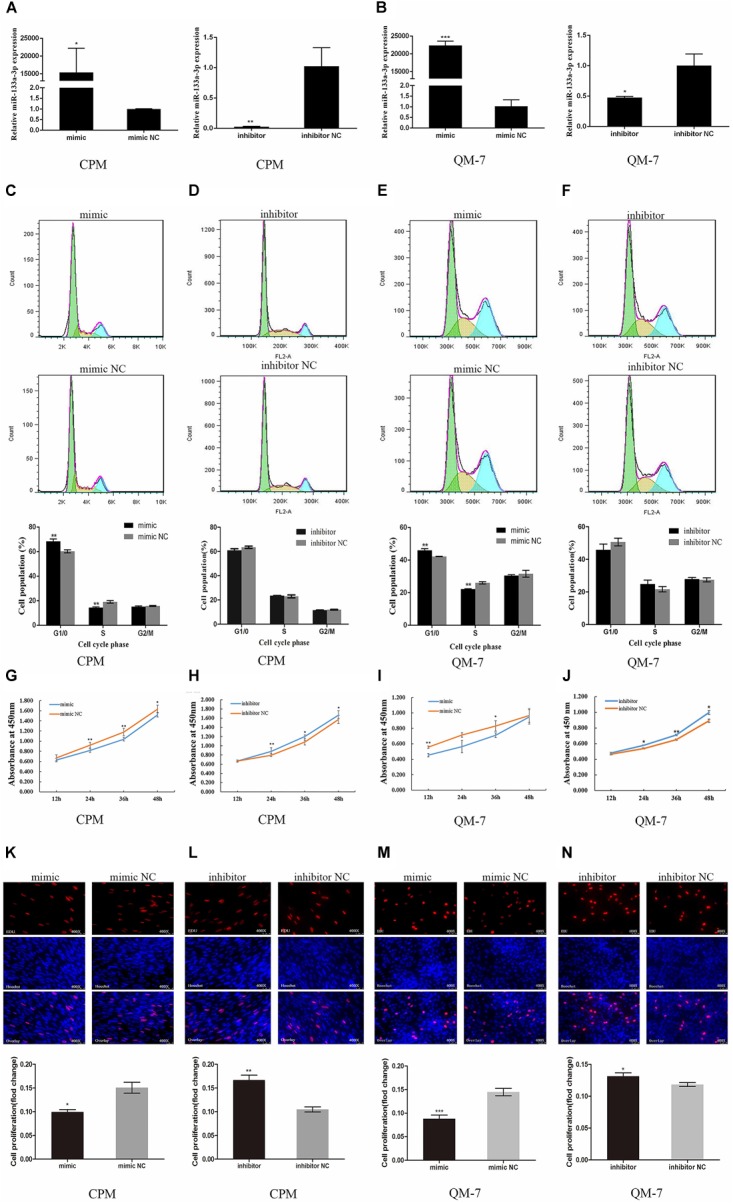
gga-mir-133a-3p inhibits myoblast proliferation. **(A,B)** Overexpression and inhibitory effects of gga-mir-133a-3p mimic and inhibitor in CPMs and QM-7 cells. **(C,E)** Cell cycle after overexpression of gga-mir-133a-3p in CPMs and QM-7 cells. **(D,F)** Cell cycle after inhibition of gga-mir-133a-3p in CPMs and QM-7 cells. **(G–J)** Cell growth curves were measured following the transfection of gga-mir-133a-3p mimic and inhibitor in CPMs and QM-7 cells. **(K,M)** Evaluation of the proliferation of gga-mir-133a-3p mimic-transfected CPMs and QM-7 cells by EdU incorporation, EdU-stained cell proportions were counted. **(L,N)** Evaluation of the proliferation of gga-mir-133a-3p inhibitor-transfected CPMs and QM-7 cells by EdU incorporation, EdU-stained cell proportions were counted. The results of all groups are shown as mean ± S.E.M., and the data represent three independent assessment methods. Statistical significance of the mean difference was assessed using an unpaired two-sample *t*-tests (^∗^*p* < 0.05; ^∗∗^*p* < 0.01; ^∗∗∗^*p* < 0.001) vs. NC (negative control).

To verify the regulation of gga-mir-133a-3p in the proliferation and differentiation of chicken myoblasts, we synthesized gga-mir-133a-3p mimic and inhibitor for the over-expression and suppression of gga-mir-133a-3p at the cellular level. First, we transfected gga-mir-133a-3p mimic and inhibitor in QM-7 cells and CPMs to observe the over-expression effect of mimic and the inhibitory effect of inhibitor for gga-mir-133a-3p. The results showed that the mimic could significantly overexpress gga-mir-133a-3p, and the inhibitor also has significant inhibitory effect for gga-mir-133a-3p (Figures 1A,B), proved that they can be used for the next verification experiments.

We performed flow cytometric experiment to examine the effect of gga-mir-133a-3p in the cell cycle of QM-7 cells and CPMs. We found that the proportion of G1 phase cells in CPMs was significantly higher than the control group (*p* < 0.01), while the proportion of cells in S phase was significantly lower than that of the control group (*p* < 0.01) after the overexpression of gga-mir-133a-3p (Figure 1C). The number of cells in the G1 phase was significantly increased (*p* < 0.01), when the overexpressed of gga-mir-133a-3p in QM-7 cells, and the number of cells in the S phase was significantly decreased (*p* < 0.01) (Figure 1E). In contrast, after the transfection of gga-mir-133a-3p inhibitor, the results showed the opposite trend effect (Figures 1D,F). The results of cell cycle assay showed that gga-mir-133a-3p plays an inhibitory effect on the cell cycle progression of CPMs and QM-7 cells.

CCK-8 assay was used for the detection of proliferation vitality in CPMs and QM-7 cells, and the results showed that the proliferation vitality of the cells which had been transfected with gga-mir-133a-3p mimic were significantly lower than that of the control (Figures 1G,I). After the inhibition of gga-mir-133a-3p, the proliferation vitality of cells was significantly increased (Figures 1H,J). It indicates that gga-mir-133a-3p can effectively inhibit the proliferation vitality of myoblasts.

We also conducted EdU experiment to determine the proportion of proliferating cells of myoblasts. The EdU experiment showed that the overexpression of gga-mir-133a-3p in CPMs can significantly decrease the number of the cells in proliferation period (*p* < 0.05), in other hand, the number of cells in proliferation phase was extremely increased with the treatment of gga-mir-133a-3p inhibitor (*p* < 0.01) (Figures 1K,L). There is also a similar effect on the QM-7 cells (Figures 1M,N). These results fully demonstrate that gga-mir-133a-3p can inhibit the proliferating of myoblasts.

### gga-mir-133a-3p Promotes Myoblast Differentiation

**FIGURE 2:**
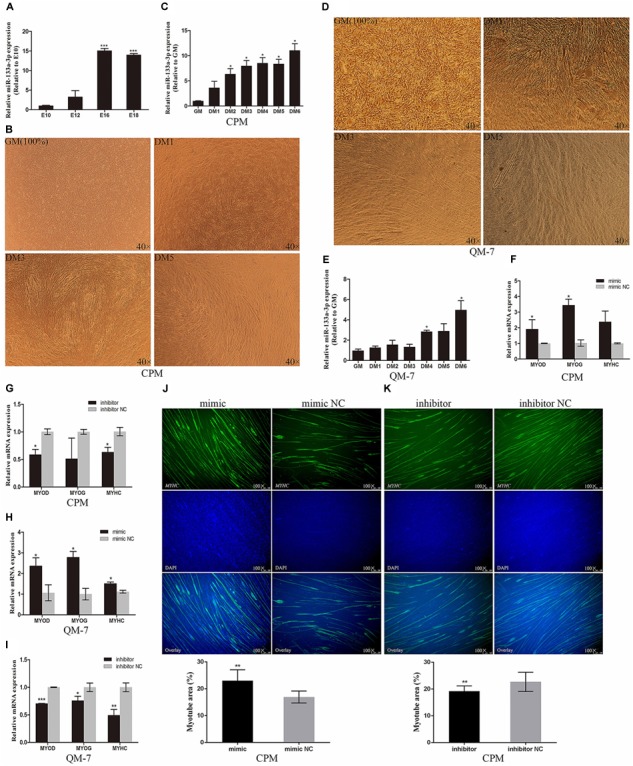
gga-mir-133a-3p promotes myoblast differentiation. **(A)** The relative expression of gga-mir-133a-3p in chicken embryonic leg muscle. **(B)** Morphology of CPMs induced differentiation. **(C)** The expression of gga-mir-133a-3p in the process of CPMs induced differentiation. **(D)** Morphology of QM-7 cells induced differentiation. **(E)** The expression of gga-mir-133a-3p in the process of QM-7 cells induced differentiation. **(F,H)** The expression of *MYOD*, *MYOG* and *MYHC* in CPMs and QM-7 cells after overexpression of gga-mir-133a-3p. **(G,I)** The expression of *MYOD*, *MYOG* and *MYHC* in CPMs and QM-7 cells after inhibition of gga-mir-133a-3p. **(J,K)** Immunofluorescence of *MyHC* and comparison of the area of myotubes. The results of all groups are shown as mean ± S.E.M., and the data represent three independent assessment methods. Statistical significance of the mean difference was assessed using an unpaired two-sample *t*-tests (^∗^*p* < 0.05; ^∗∗^*p* < 0.01; ^∗∗∗^*p* < 0.001) vs. NC (negative control).

To investigate the potential mechanism of gga-mir-133a-3p in myogenesis of chicken skeletal muscle during the embryonic period, hence we isolated leg muscles of XingHua chicken embryo. During the period of skeletal muscle development in embryo of XingHua chicken, the expression of gga-mir-133a-3p in E16 and E18 were significantly up-regulated relative to E10 and E12 (Figure 2A), which suggests that gga-mir-133a-3p may be related to the differentiation of skeletal myoblasts. To further verify the potential role of gga-mir-133a-3p, CPMs were induced to differentiate *in vitro* (Figure 2B). With the process of differentiation in CPMs, the expression of gga-mir-133a-3p was significantly up-regulated, which suggest that gga-mir-133a-3p may be involved in the regulation of differentiation of myoblasts (Figure 2C). We also performed the same experiment in QM-7 cells, and similar results have been confirmed (Figures 2D,E).

Differentiation of myoblasts is accompanied by an increase in the expression of myogenic differentiation marker genes, including *MYOD*, *MYOG*, and *MYHC*. Here further determine of the relationship between gga-mir-133a-3p and differentiation of myoblasts, we performed the overexpression and the inhibition of gga-mir-133a-3p in CPMs and QM-7 cells, and then, induced cells differentiation. The results showed that gga-mir-133a-3p can significantly increase the expression of myogenic differentiation marker genes in CPMs (Figures 2F,G). And the same effect has shown in QM-7 cells (Figures 2H,I).

Using immunofluorescence, we traced the effects of gga-mir-133a-3p on myoblast differentiation. After transfecting gga-mir-133a-3p mimics, the area of myotubes formed by cell fusion was larger relative to the control group (Figure 2J), while the area of the myotube formed by cell fusion was smaller than that of the control group after the transfection of gga-mir-133a-3p inhibitor (Figure 2K). The results show that gga-mir-133a-3p play a promotional effect on myoblast differentiation.

### gga-mir-133a-3p Targets Directly *PRRX1* Gene

In order to explore the regulatory mechanism of gga-mir-133a-3p in growth and development of chicken embryo skeletal muscle and how gga-mir-133a-3p regulates the proliferation and differentiation of skeletal muscle myoblasts, we tried to determine the target genes of gga-mir-133a-3p related to the proliferation and differentiation of skeletal muscle myoblasts. The literature has confirmed that the functions of miRNAs are regulating the expression of its target genes.

**FIGURE 3:**
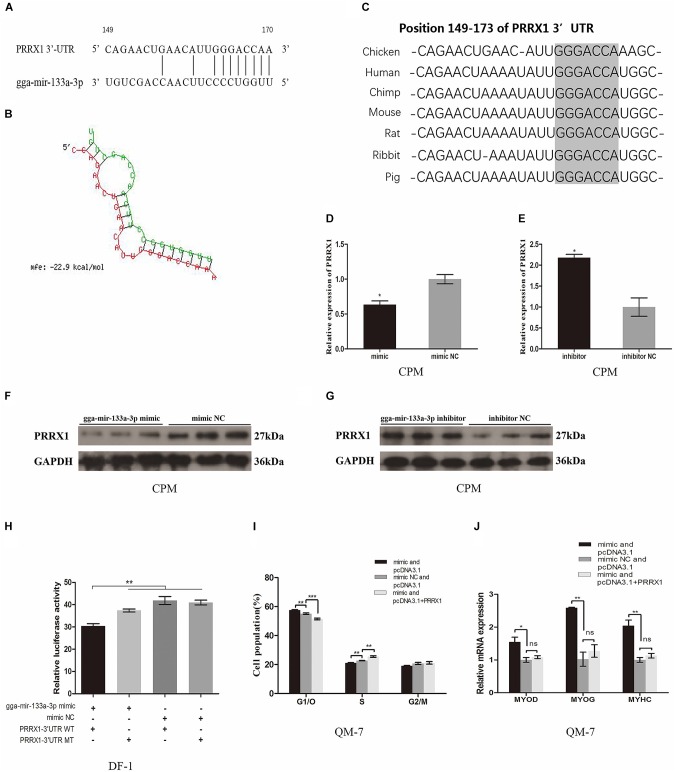
gga-mir-133a-3p targets directly *PRRX1* gene. **(A)** The potential binding sites of gga-mir-133a-3p in *PRRX1* 3′UTR. **(B)** Duplex and MFE between gga-mir-133a-3p and *PRRX1* 3′UTR were analyzed by RNAhybrid. **(C)** Interspecies conservation in seed binding regions. **(D,E)** The expression of *PRRX1* in CPMs after overexpression and inhibition of gga-mir-133a-3p. **(F,G)** The protein expression of *PRRX1* in CPMs after overexpression and inhibition of gga-mir-133a-3p. **(H)** Luciferase assay was conducted by co-transfecting wild type or mutant *PRRX1* 3′UTR with gga-mir-133a-3p mimic or mimic NC in DF-1 cells. **(I)** Cell cycle analysis of QM-7 cells after co-transfection with the listed nucleic acids. **(J)** The mRNA expression levels of myoblast differentiation marker genes from QM-7 cells after co-transfection. The results of all groups are shown as mean ± S.E.M., and the data represent three independent assessment methods. Statistical significance of the mean difference was assessed using an unpaired two-sample *t*-tests (^∗^*p* < 0.05; ^∗∗^*p* < 0.01; ^∗∗∗^*p* < 0.001) vs. NC (negative control).

The miRDB was used to predict and search the possible target genes of gga-mir-133a-3p. Data collection and analysis showed that *PRRX1* gene was a potential target gene for gga-mir-133a-3p (Target Score: 99). In our previous study, the expression level of *PRRX1* was down-regulated in the leg muscles between the E11 and E16 XingHua chickens, which were opposite to the expression level of gga-mir-133a-3p. The predicted binding site is at position 149–170 of the 3′UTR and the seed binding sequence is located at position 163–169 of the 3′UTR (Figure 3A). Besides, the RNAhybrid was used to analyze the duplex and the MFE between gga-mir-133a-3p and *PRRX1* 3′UTR. The RNA duplex has MFE approximately -22.9 kcal/mol, which indicating that more stable state (Figure 3B). The predicted target binding site at the 3′UTR of *PRRX1* is conserved among species, including human, chimp, mouse, rat, rabbit and pig (Figure 3C).

To verify the possible target relationship between gga-mir-133a-3p and *PRRX1* in myoblasts, we overexpressed and inhibited gga-mir-133a-3p in CPMs, and then, quantified the expression of *PRRX1* by RT-PCR and western blot. The results showed that gga-mir-133a-3p can curb the expression of *PRRX1* both in the level of mRNA and protein (Figures 3D–G).

To further verify the target relationship between gga-mir-133a-3p and *PRRX1*, we performed a dual-luciferase reporter assay. The 3′UTR target sequence was cloned into the luciferase reporter vector (pmirGLO-*PRRX1*-3′UTR WT) and a luciferase reporter vector was constructed that mutated the target sequence binding site (pmirGLO-*PRRX1*-3′UTR MT). We transfected pmirGLO-*PRRX1*-3′UTR WT or pmirGLO-*PRRX1*-3′UTR MT with the gga-mir-133a-3p mimic or mimic NC into the DF-1 cells, respectively, and detected the luciferase activity after 48 h. Expression of luciferase can be inhibited by binding of gga-mir-133a-3p to 3′UTR of *PRRX1*. The luciferase activity was significantly decreased after co-transfection of gga-mir-133a-3p and *PRRX1*-3′UTR WT (Figure 3H).

To evaluate the roles of *PRRX1* in the biological action of gga-mir-133a-3p, we conducted a recovery validation experiment of gga-mir-133a-3p function by co-transfecting with: gga-mir-133a-3p mimic and pcDNA3.1, mimic NC and pcDNA3.1, gga-mir-133a-3p mimic and pcDNA3.1+*PRRX1* to test their effects on myoblast proliferation and differentiation. The results have shown that gga-mir-133a-3p can inhibit the proliferation of myoblasts, upregulate the expression of differentiation marker genes. However, these effects on myoblast proliferation and differentiation were restored or even reversed by co-expression of gga-mir-133a-3p and *PRRX1* (Figures 3I,J). These results sufficiently indicated a direct target relationship between gga-mir-133a-3p and *PRRX1*.

### *PRRX1* Promotes Myoblast Proliferation

**FIGURE 4:**
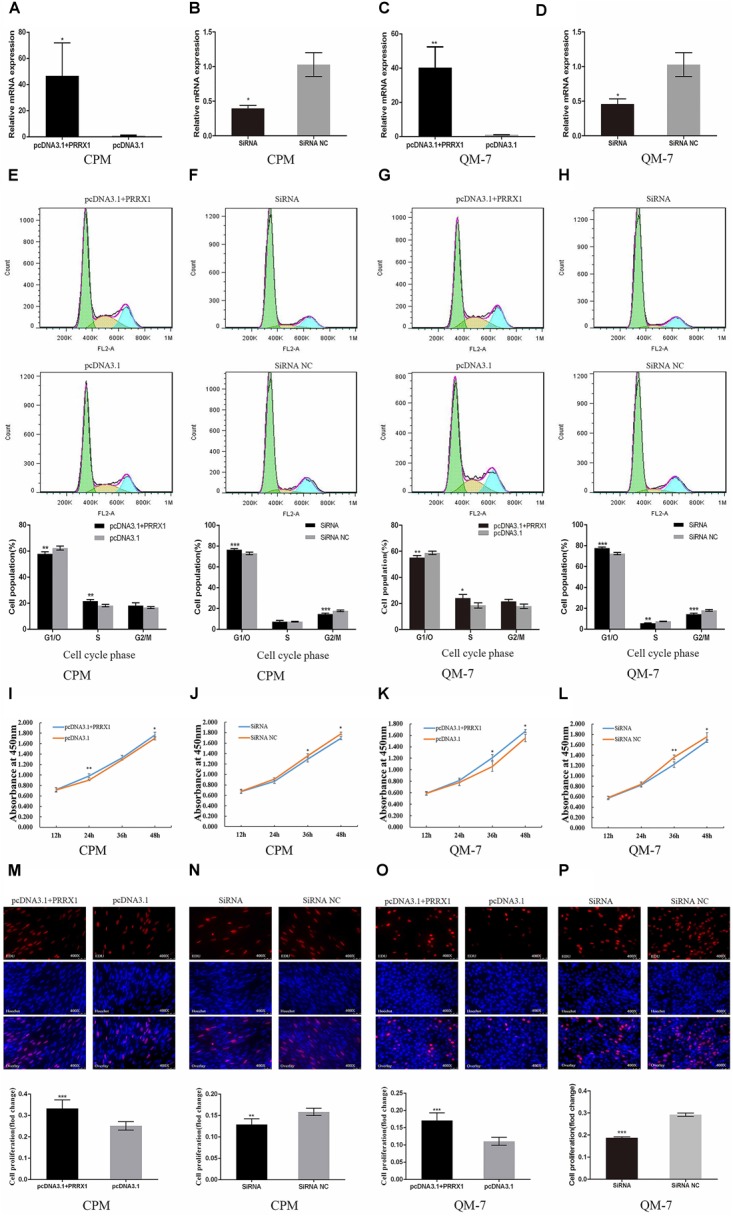
*PRRX1* promotes myoblast proliferation. **(A,C)** The overexpression efficiency of the pcDNA3.1 with *PRRX1* in CPMs and QM-7 cells. **(B,D)** The interference efficiency of *PRRX1* SiRNA in CPMs and QM-7 cells. **(E,G)** Cell cycle after overexpression of *PRRX1* in CPMs and QM-7 cells. **(F,H)** Cell cycle after interference of *PRRX1* in CPMs and QM-7 cells. **(I–L)** Cell growth curves were measured following the transfection of *PRRX1* overexpression vector and SiRNA in CPMs and QM-7 cells. **(M,O)** Evaluation of the proliferation of *PRRX1*-transfected CPMs and QM-7 cells by EdU incorporation, EdU-stained cell proportions were counted. **(N,P)** Evaluation of the proliferation of *PRRX1* SiRNA-transfected CPMs and QM-7 cells by EdU incorporation, EdU-stained cell proportions were counted. The results of all groups are shown as mean ± S.E.M., and the data represent three independent assessment methods. Statistical significance of the mean difference was assessed using an unpaired two-sample *t*-tests (^∗^*p* < 0.05; ^∗∗^*p* < 0.01; ^∗∗∗^*p* < 0.001) vs. NC (negative control).

We constructed the overexpression vector of *PRRX1* gene and synthesized its siRNA, to determine whether *PRRX1* regulates the proliferation and differentiation of myoblasts. And their over-expression efficiency and interference efficiency were determined in CPMs and QM-7 cells, respectively (Figures 4A–D).

Flow cytometry results showed that overexpression of *PRRX1* in CPMs can significantly reduce the proportion of cells in G1/0 phase and increase the proportion of cells in S phase, contrary to the results after siRNA treatment (Figures 4E,F). We also got similar results after conducting the same test in QM-7 cells (Figures 4G,H). The results indicate that *PRRX1* can effectively promote the cycle progression of myoblasts.

Similarly, we examined the proliferation viability of myoblasts through CCK-8. After the treatment of overexpression of *PRRX1* in CPMs, the proliferation viability of cells has been significantly increased (Figure 4I). Interfering with *PRRX1* has reduced the viability of cells (Figure 4J). The results were consistent with that of the experiments in QM-7 cells (Figures 4K,L). The experimental results demonstrate that *PRRX1* can enhance the proliferation activity of myoblasts.

Higher and fewer proliferating cells were detected after overexpression and interference with *PRRX1* in CPMs, respectively (Figures 4M–P), which indicating that *PRRX1* can promote CPMs and QM-7 cells proliferation.

### *PRRX1* Suppresses Myoblast Differentiation

**FIGURE 5:**
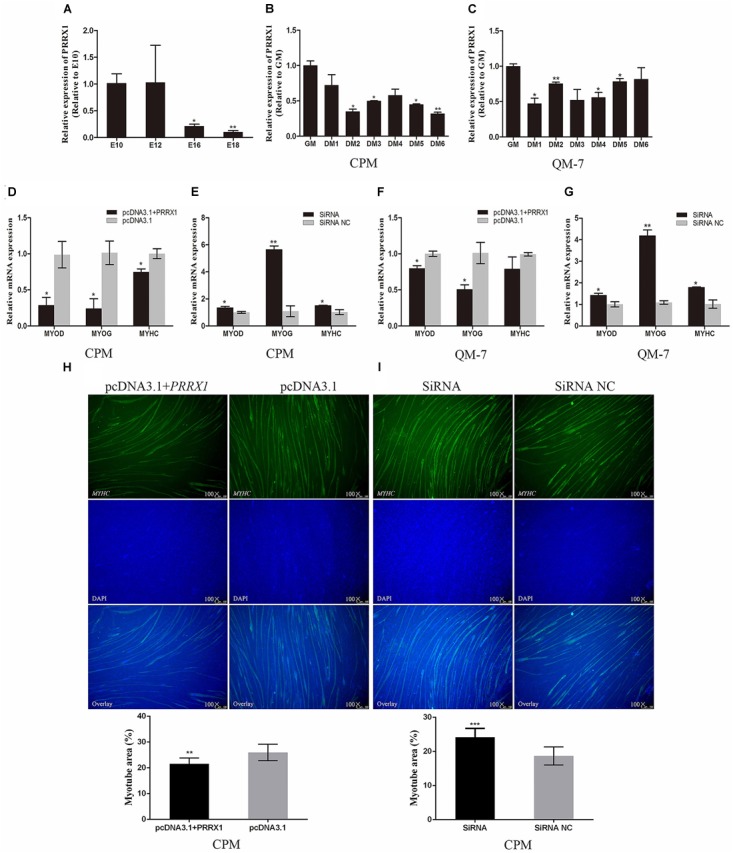
*PRRX1* suppresses myoblast differentiation. **(A)** The relative expression of *PRRX1* in chicken embryonic leg muscle. **(B)** The expression of *PRRX1* in the process of CPMs induced differentiation. **(C)** The expression of *PRRX1* in the process of QM-7 cells induced differentiation. **(D,F)** The expression of *MYOD*, *MYOG*, and *MYHC* in CPMs and QM-7 cells after overexpression of *PRRX1*. **(E,G)** The expression of *MYOD*, *MYOG*, and *MYHC* in CPMs and QM-7 cells after interference of *PRRX1*. **(H,I)** Immunofluorescence of *MyHC* and comparison of the area of myotubes. The results of all groups are shown as mean ± S.E.M., and the data represent three independent assessment methods. Statistical significance of the mean difference was assessed using an unpaired two-sample *t*-tests (^∗^*p* < 0.05; ^∗∗^*p* < 0.01; ^∗∗∗^*p* < 0.001) vs. NC (negative control).

During the period of skeletal muscle development in embryo of XingHua chicken, the expression of *PRRX1* in E16 and E18 were significantly down-regulated relative to E10 (Figure 5A), which shows that *PRRX1* may be related to the differentiation of skeletal myoblasts.

After CPMs and QM-7 cells were induced to differentiate, we performed RT-PCR to quantify the expression level of *PRRX1*. The expression of *PRRX1* in the differentiation phase is lower than in the proliferative phase in CPMs and QM-7 cells (Figures 5B,C). *PRRX1* can significantly decrease the expression level of myogenic marker genes related to the differentiation of myoblasts, including *MYOD*, *MYOG*, *MYHC* (Figures 5D,F). After interference treatment to *PRRX1*, the expression level of the myogenic marker genes was significantly up-regulated (Figures 5E,G).

In immunofluorescence experiments, the area of cell-fused myotubes was smaller than that of the control group after over-expressing *PRRX1*, while the area of cell-fused myotubes was larger relative to the control group after silencing *PRRX1* (Figures 5H,I). The results indicated that *PRRX1* has an inhibitory effect on myoblast differentiation.

## Discussion

In this study, we revealed a role of gga-mir-133a-3p in the proliferation and differentiation of chicken myoblasts, and we also found it is based on the function of regulating the proliferation and differentiation of myoblasts by inhibiting *PRRX1* (Supplementary Figure S1). The proliferation and differentiation of skeletal muscle cells were relatively independent and antagonistic process, and miRNAs can regulate this process and control the proliferation and differentiation of myoblasts. Studies have also shown that mir-206 and mir-486 can accelerate muscle cell proliferation from the proliferative phase to the differentiation phase by inhibiting the expression of *PAX7* (Dey et al., 2011). Mir-133a-3p is associated with proliferation and differentiation of embryonic muscle cells, as well as oral squamous cell carcinoma (Lewis et al., 2005). In our current study, gga-mir-133a-3p is differentially expressed between the leg muscles of XingHua chickens at embryonic E11 and E16. In our study, we found the transient expression pattern of gga-mir-133a-3p during skeletal muscle development and it was upregulated, which suggests that, a potential role in skeletal muscle myogenesis. To verify the potential role of gga-mir-133a-3p for skeletal muscle development, we performed experiments on the proliferation of CPMs and QM-7 cells. We confirmed that gga-mir-133a-3p can inhibit the proliferation of myoblasts. In other hand, we performed a series of experiments to confirm that gga-mir-133a-3p can promote myoblast differentiation. Mir-133 participated in myoblasts proliferation and differentiation by regulating the negatively modulates *IGF-1R/PI3K/Akt* signaling through repression of *IGF-1R* (Huang et al., 2011). The study completed by Chen et al. indicated that mir-133 can enhance myoblast proliferation (Chen et al., 2006), which is inconsistent with our results. However, they did research in mouse myoblasts and we performed that the effect of gga-mir-133a-3p on the growth and development of chicken skeletal muscle. In addition, *in vitro* study of Chen et al. overexpressed mir-133 using an overexpression vector inserted with a precursor sequence of mir-133 to verify *pho-H3* expression in C2C12 cells, which they concluded that mir-133 enhances myoblast proliferation without further validation experiments. Our study indicated that the chicken mir-133 inhibits myoblast proliferation with more evidence supported by flow cytometric analysis, CCK-8 assay as well as EdU assay. These may be the reason why our results are contrasting with Chen et al. (2006) studied. It has been demonstrated that mir-133 inhibits cyclin D1 expression and induces myoblast G1 arrest, thereby inhibiting myoblast proliferation (Zhang et al., 2012). Another study has also demonstrated that mir-133 inhibits myoblast proliferation and promotes myoblast differentiation by repressing the activity of the *ERK1/2* pathway through targeting *FGFR1* and *PP2AC* (Feng et al., 2013).

MiRNAs mainly exert their biological effects by regulating the expression of target genes by binding to the 3′UTR of their target genes (Lewis et al., 2005). We performed target gene prediction analysis on the miRDB website. 131 genes were predicted and the top 10 scored genes were listed (Supplementary Table S1). These genes are mainly involved a variety of biological functions, including post-transcriptional splicing, DNA repair, mRNA maturation and so on, which also play a role in multiple biological processes, such as limb growth, muscle development, cellular immunity. We found that the mature sequence of the gga-mir-133a-3p can match the 3′UTR of *PRRX1*, which was downregulated between the leg muscles of E11 and E16 in XingHua chickens. Through data analysis, we found that the 3′UTR of *PRRX1* and mir-133a-3p can be stably combined, and its seed binding area is conserved between multiple species. Comparing the conservation of the seed sequence of mir-133a-3p among these species, we found that the seed sequence of pig and chicken is exactly the same, and the other several species have only 85.71% homology with chicken (Supplementary Figure S2). It was shown that gga-mir-133a-3p and ssc-miR-133a-3p are specific for the targeted binding of *PRRX1* relative to the other species. The result of dual luciferase assay demonstrated the direct targeting relationship between gga-mir-133a-3p and *PRRX1*. After overexpression and inhibition of gga-mir-133a-3p, the expression of *PRRX1* was also decreased and increased in the levels of transcription and translation, respectively.

*PRRX1* is known as a transcription factor, and its temporal and spatial expression are involved in the differentiation of cells and the development of tissues and organs (Higuchi et al., 2013). Previous studies have reported that the *PRRX1* gene is involved in cell proliferation and reorganization (White et al., 2003). *PRRX1* is localized to differentiate endothelial cells in the fetal lung mesenchyme and is critical for pulmonary angiogenesis (Ihida-Stansbury et al., 2004). At present, the research on *PRRX1* mainly focused on embryonic limb development, vascular smooth muscle differentiation and osteogenic differentiation (Martin et al., 1995; Ten et al., 1998; Cretekos et al., 2008; Shang et al., 2008). In addition, *PRRX1*, was shown decreasing expression during chicken skeletal muscle development (Huang et al., 2011; Higuchi et al., 2013; He et al., 2018), which indicated that potential role in skeletal muscle growth and development. CCK-8 assay, EdU assay and flow cytometry were used to detect the effect of *PRRX1* in myoblast proliferation. A series of *in vitro* experiments have demonstrated that *PRRX1* can significantly promote the proliferation of myoblasts. At the same time, *PRRX1* is also down-expressed during myoblast differentiation, which revealed that *PRRX1* may be related to differentiation of myoblasts. The overexpression of *PRRX1* can significantly reduce the expression of myogenic factors which were related to myoblast differentiation, including *MYOD*, *MYOG*, and *MYHC*. And overexpression of *PRRX1* can inhibit myotube formation. It indicated that *PRRX1* can suppress myoblast differentiation.

In summary, our study revealed a novel mechanism of gga-mir-133a-3p in regulating skeletal muscle growth and development. Gga-mir-133a-3p can regulate myogenesis by inhibiting myoblast proliferation and promoting myoblast differentiation, and it exerts biological effects through targeted inhibition of *PRRX1* expression.

## Author Contributions

LG conducted most of experiments, data analysis, and drafted the manuscripts. WH and BC performed parts of verification experiments and manuscript writing. EJB was responsible for the English editing of the article and part of experiments. BC and XC provided experimental ideas. QN participated in the design of the whole study. All authors read and approved the final manuscript.

## Conflict of Interest Statement

The authors declare that the research was conducted in the absence of any commercial or financial relationships that could be construed as a potential conflict of interest.

## Footnotes

1 http://mirdb.org

2 https://bibiserv.cebitec.uni-bielefeld.de/rnahybrid

## Supplementary Material

FIGURE S1 Model of gga-mir-133a-3p-mediated regulatory mechanism for myoblast proliferation and differentiation. In simple terms, gga-mir-133a-3p inhibits the expression level of both *PRRX1* mRNA and protein by targeting the 3 ’UTR of *PRRX1* gene, resulting in the inhibition of myoblasts proliferation but promoting myoblasts differentiation.

FIGURE S2 Comparison of the seed sequence of mir-133a-3p among different species, including chicken, human, mouse, rat, rabbit and pig.

TABLE S1 Top 10 target gene scores for gga-mir-133a-3p as predicted using the miRNA target prediction and functional annotations miRDB tool (URL: http://www.mirdb.org/).
