# Supplementary figures and images for: gga-mir-133a-3p Regulates Myoblasts Proliferation and Differentiation by Targeting PRRX1
Source: Front Genet. 2018 Dec 4;9:577. doi: 10.3389/fgene.2018.00577 (PMC6288258; doi:10.3389/fgene.2018.00577)

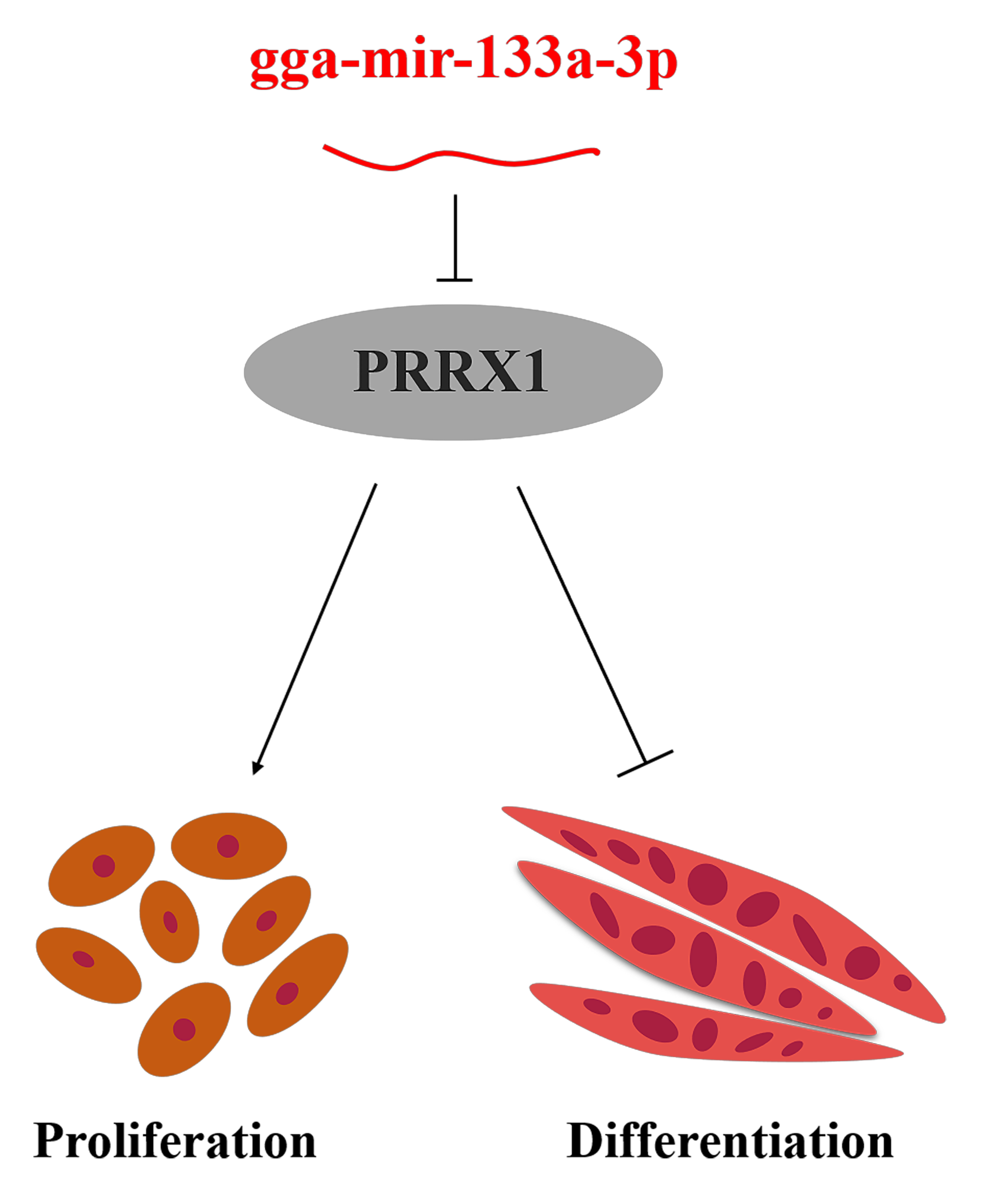

Supplement: FIGURE S1 — Model of gga-mir-133a-3p-mediated regulatory mechanism for myoblast proliferation and differentiation. In simple terms, gga-mir-133a-3p inhibits the expression level of both PRRX1 mRNA and protein by targeting the 3 ’UTR of PRRX1 gene, resulting in the inhibition of myoblasts proliferation but promoting myoblasts differentiation. [file Image_1.TIF]

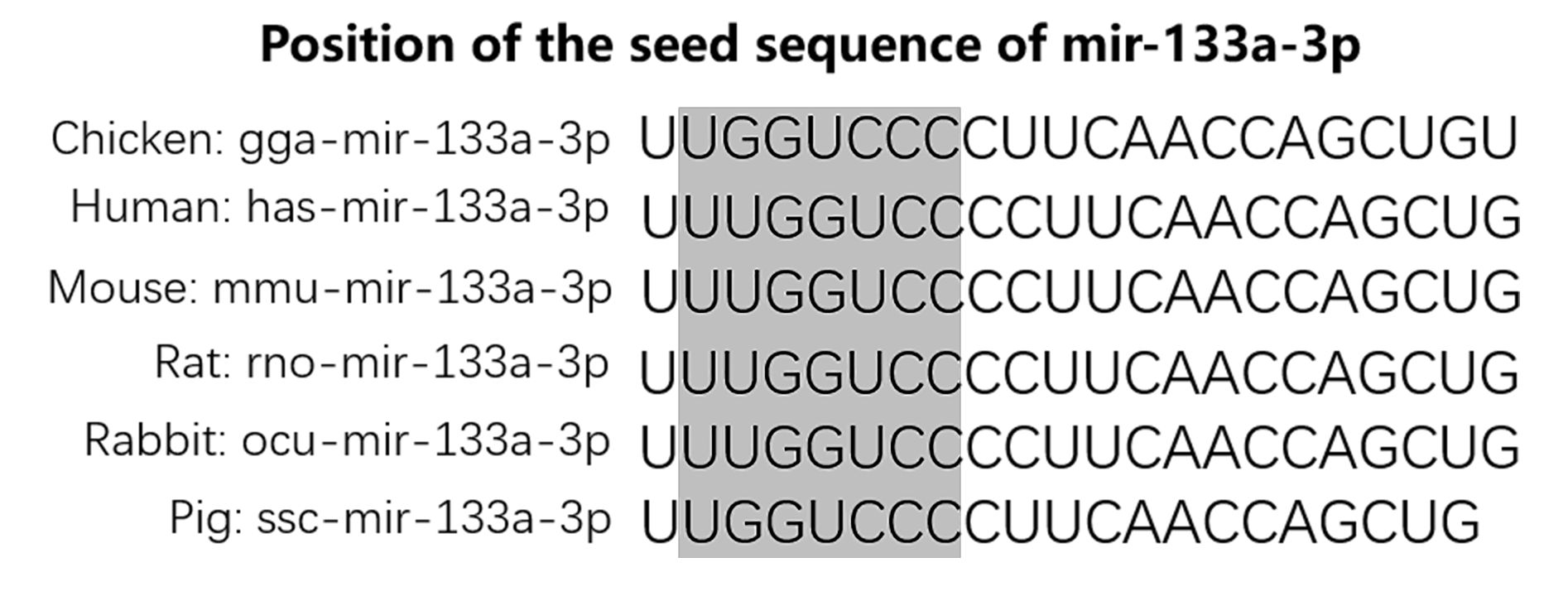

Supplement: FIGURE S2 — Comparison of the seed sequence of mir-133a-3p among different species, including chicken, human, mouse, rat, rabbit and pig. [file Image_2.TIF]
