# Supplementary material for: gga-mir-133a-3p Regulates Myoblasts Proliferation and Differentiation by Targeting PRRX1
Source: Front Genet. 2018 Dec 4;9:577. doi: 10.3389/fgene.2018.00577 (PMC6288258; doi:10.3389/fgene.2018.00577)
Supplement: TABLE S1 — Top 10 target gene scores for gga-mir-133a-3p as predicted using the miRNA target prediction and functional annotations miRDB tool (URL: http://www.mirdb.org/). [file Table_1.docx]

Supplementary Material

gga-mir-133a-3p regulates myoblasts proliferation and differentiation by targeting *PRRX1*

Lijin Guo, Weiling Huang, Biao Chen, Endashaw Jebessa, Xiaolan Chen, Bolin Cai, and Qinghua Nie^*^

*** Correspondence:** Qinghua Nie: [nqinghua@scau.edu.cn](mailto:nqinghua@scau.edu.cn)

# Supplementary Table 1

| **Target Rank** | **Target Score** | **miRNA Name** | **Gene Symbol** | **Gene Description** |
| --- | --- | --- | --- | --- |
| 1 | 99 | gga-mir-133a-3p | *PRRX1* | paired related homeobox 1 |
| 2 | 98 | gga-mir-133a-3p | *PTBP2* | polypyrimidine tract binding protein 2 |
| 3 | 98 | gga-mir-133a-3p | *SMEK1* | SMEK homolog 1, suppressor of mek1 (Dictyostelium) |
| 4 | 97 | gga-mir-133a-3p | *AKAP9* | A kinase (PRKA) anchor protein (yotiao) 9 |
| 5 | 96 | gga-mir-133a-3p | *SMEK2* | SMEK homolog 2, suppressor of mek1 (Dictyostelium) |
| 6 | 96 | gga-mir-133a-3p | *PREX1* | phosphatidylinositol-3,4,5-trisphosphate-dependent Rac exchange factor 1 |
| 7 | 95 | gga-mir-133a-3p | *CELF4* | CUGBP, Elav-like family member 4 |
| 8 | 95 | gga-mir-133a-3p | *CYB5B* | outer mitochondrial membrane cytochrome b5 |
| 9 | 94 | gga-mir-133a-3p | *DDX3X* | DEAD (Asp-Glu-Ala-Asp) box polypeptide 3, X-linked |
| 10 | 94 | gga-mir-133a-3p | *C6H10ORF46* | chromosome 6 open reading frame, human C10orf46 |

**Supplementary Table 1.** **Top 10 target gene scores for gga-mir-133a-3p as predicted using the miRNA target prediction and functional annotations miRDB tool (URL: http://www.mirdb.org/).**
